# A Review of Polyphenolics in Oak Woods

**DOI:** 10.3390/ijms16046978

**Published:** 2015-03-27

**Authors:** Bo Zhang, Jian Cai, Chang-Qing Duan, Malcolm J. Reeves, Fei He

**Affiliations:** 1 Center for Viticulture and Enology, College of Food Science and Nutritional Engineering, China Agricultural University, Beijing 100083, China; E-Mails: zb692002@163.com (B.Z.); caijian928@cau.edu.cn (J.C.); chqduan@cau.edu.cn (C.-Q.D.); mreeves@xtra.co.nz (M.J.R.); 2 College of Food Science and Engineering, Gansu Agricultural University, Lanzhou 730070, China; 3 College of Agronomy and Biotechnology, China Agricultural University, Beijing 100094, China; 4 Institute of Food, Nutrition and Human Health, Massey University, Palmerston North 4442, New Zealand

**Keywords:** oak, polyphenolics, structure, wine aging, analysis

## Abstract

Polyphenolics, which are ubiquitous in plants, currently are among the most studied phytochemicals because of their perceptible chemical properties and antioxidant activity. Oak barrels and their alternatives, which are widely used in winemaking nowadays, contribute polyphenolics to wines and are thought to play crucial roles in the development of wines during aging. This study summarizes the detailed information of polyphenolics in oak woods and their products by examining their structures and discussing their chemical reactions during wine aging. This paper evaluates the most recent developments in polyphenolic chemistry by summarizing their extraction, separation, and their identification by the use of chromatographic and spectral techniques. In addition, this paper also introduces polyphenol bioactive ingredients in other plant foods.

## 1. Introduction

It was possibly the Celts who invented wooden barrels that were nearly identical to the basic forms used today. In the third century B.C., these barrels gradually replaced amphoras as the main containers for wine transportation [1]. Ancient Mesopotamians initially used barrels made of palm wood, although not for wine. However, it is difficult to bend and fashion palm wood into barrels and so their use was limited, especially for liquids. With the various limitations of palm wood, wine merchants in different regions experimented with various wood sources to find the better materials for barrel making [2]. The use of oak for barrel production had been prevalent for about 2000 years, and the earliest literature on use of oak can be traced back to the Roman Empire. With the passage of time, winemakers discovered that wine aging in oak barrels was not only convenient, but also improved wine quality by improving their appearances, flavors and mouth feel, therefore, aging wines in oak barrels became an indispensable part of making high-quality wines [2,3,4]. Given the unique mechanical, physical, and chemical properties of oak wood, especially oak heartwood, it emerged as being particularly suitable not only for shipping wine but also for its development with time.

Oak is an angiosperm belonging to the Fagacea family. Approximately 600 species of the genus *Quercus* are distributed worldwide, and the basic composition of oak does not significantly differ from one species to another. Cellulose (40%) and hemicellulose (25%), which provide the framework and matrix of the woods, are the main compounds of oak woods, and lignin, another large polymer mainly present in the cell walls, also comprises 20% of dried oak wood [5,6].

American white oak (*Q. alba*) and French red oak (*Q. robur* and *Q. petraea*) are three of the most frequently used sources of oak woods in coopering [7]. Some previous studies also focused on examining new types and new regions of oak wood from Spain, Russia and other Eastern Europe countries (Ukraine, Romania, Hungary), even in China, with a view to sustaining the existing oak resources by finding suitable new alternatives and suppliers [8,9,10]. Relevant studies showed that wines aged in Spanish oak barrels had characteristics similar to those of the same wines aged in French oak barrels. However, the wines aged in American oak barrels usually showed significant differences compared with the wines aged in Spanish counterparts. Some results suggested that Eastern Europe oak contained higher levels of aromatic substances, such as volatile phenols and phenolic aldehydes, than French oak, even though they were of the same species [10,11]. These findings demonstrated the prospects of new oak sources for the wine industry, and such sources might soon compete with traditional ones.

Generally speaking, oak barrel aging of wines is considered as a traditional and standard part of winemaking to yield high-quality wines. During aging, various soluble oak components diffuse into the wine and enhance the intensity and complexity of wine flavors [11]. Volatile phenols and benzoic aldehydes play a very important role in contributing to the sensorial characteristics of the wines [12,13]. Hydrolyzable tannins such as ellagitannins, are particularly significant because they confer astringency, as well as being involved in the stabilization of pigment structures [14]. Moreover, during aging, micro-amounts of oxygen penetrate into the wine due to the slightly porous nature of the oak grain. This slow infusion improves the quality of red wines, especially in terms of color, aroma, and taste [15,16,17,18,19]. Thus, during aging in oak barrels, wines acquire better color, more delicate aromas and greater roundness, improve their harmony in taste profile, and decrease the intensities of green and woody characters. Furthermore, the aging process is not only a critical step in improving the sensory properties of wines but also in acquiring health protective properties. These properties include an increase in antioxidant activity, anticarcinogenic properties, and free radical scavenging capacity [20,21,22].

Such benefits have stimulated the considerable researches into the roles of oak in wine aging over several decades. Many studies focused on the composition and function of oak, and how it contributed to improve wine quality. Therefore, the present review covers the following: (1) The basic structures of plant polyphenolic components; (2) The polyphenolic compositions of different types of oak; (3) The factors that influence oak polyphenolic compositions; (4) The available methods for the extraction, separation, and characterization of the polyphenolic compounds in oak woods and other plant sources with similar polyphenolic compositions; and finally (5) The polyphenol bioactive ingredients in other plant foods.

## 2. Classification of Plant Polyphenols

Polyphenolics constitute one of the most numerous and widely distributed groups of substances in the plant kingdom, currently with over 8000 known phenolic structures published [23]. These compounds can be subdivided into different classes according to the number of their phenol rings and the structural elements linked to the basic units [24]. Table 1 shows a general classification of the 21 principal structures based on the number of carbons in the molecule.

**Table 1:** Classification of phenolic compounds in plants [24].

Structure	Phenolic Class
C_6_	Simple phenolics
C_6_-C_1_	Phenolic acids and related compounds
C_6_-C_2_	Acetophenones and phenylacetic acids
C_6_-C_3_	Cinnamic acids, cinnamyl aldehydes/alcohols
C_6_-C_3_	Coumarins, isocoumarins, chromones
C_6_-C_1_-C_6_	Benzophenones, xanthones
C_6_-C_2_-C_6_	Stilbenes
C_6_-C_3_-C_6_	Chalcones, aurones, dihydrochalcones
C_6_-C_3_-C_6_	Flavones
C_6_-C_3_-C_6_	Flavonols
C_6_-C_3_-C_6_	Flavanones
C_6_-C_3_-C_6_	Flavanonols
C_6_-C_3_-C_6_	Flavan-3-ols
C_6_-C_3_-C_6_	Isoflavonoids
C_6_-C_3_-C_6_	Anthocyanidins/Anthocyanins
(C_6_-C_3_-C_6_)_2_	Biflavonoids
C_6_,C_10_,C_14_	Benzoquinones, naphthaquinones, anthraquinones
C_18_	Betacyanins
Lignans, neolignans	Dimers or oligomers
Lignin	Polymers
Phlobaphenes	Polymers

**Figure 1:**
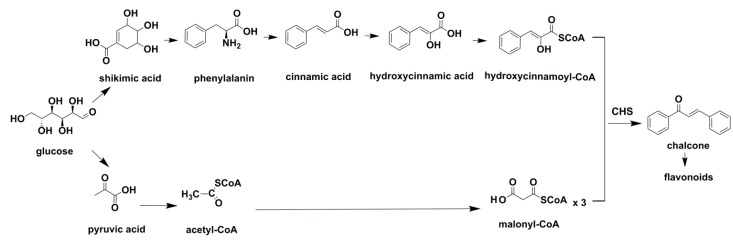
Scheme of the biosynthetic pathways of polyphenolic compounds [25].

These secondary molecules accumulate in plants and participated in defense mechanisms against the ultraviolet radiation or the aggression by pathogens. Generally, polyphenol skeletons are derived from two different active precursors (*i.e.*, 4-coumaroyl-CoA and malonyl-CoA), and they arise biogenetically from acetate and shikimate pathways (Figure 1).

Relevant studies showed that both synthetic pathways were derived from glucose metabolism. In addition, the two main precursors could be joined by a condensation reaction catalyzed by chalcone synthase (CHS) to be converted into flavonoids, which are the most abundant and important group of polyphenol compounds [26,27]. Therefore, another classification system could be established according to the flavonoid or non-flavonoid structures of the aforementioned compounds.

Flavonoid chemical skeletons are based on C15 (C_6_-C_3_-C_6_) compounds, all of which share a common diphenyl propane structure with two aromatic rings (ring A and B) joined through a group of three carbon atoms to yield an oxygenated heterocycle (ring C). Flavonoids also occasionally occur in plants as *O*- or *C*-glycosides. The preferred glycosylation sites of these compounds are the C_3_ position of the C ring, or C_5_ position of the A ring, and less frequently, the C_7_ site of flavonoid structures. Glucose is the most common sugar residue of flavonoids, but others include rhamnose, xylose, and galactose [28,29,30]. Based on experimental determination, the arrangement of C3 group determines their classification. Normally, flavonoids consist mainly of monomeric flavanols (catechins and leucoanthocyanidins), polymeric flavanols (proanthocyanidins), and chalcones, flavonols, flavanones, and anthocyanidins, which were all biosynthesized in the flavonoid pathway. Besides, isoflavonoids, biflavonoids, and neoflavanoids were also studied [31,32]. Table 2 gives the basic chemical structures of the main flavonoid compounds.

**Table 2:** The basic chemical structure of the main flavonoids compounds [31].

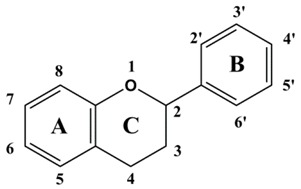

Flavonoids	Basic Structure
Chalcones	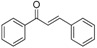
Dihydrochalcones	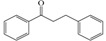
Aurones	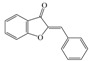
Flavonols	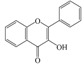
Dihydroflavonols	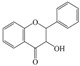
Flavanones	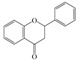
Flavanols	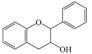
Isoflavonoids	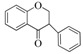
Biflavonoids	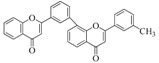
Anthocyanidins	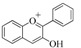

The non-flavonoid phenolic constituents in plants can be mainly classified into hydroxybenzoic acids, hydroxycinnamic acids, volatile phenols, stilbenes, and miscellaneous compounds (e.g., lignans and coumarins) [33,34]. Hydroxybenzoic acids are organic compounds that contain a phenolic ring and an associated carboxylic group, making a C_6_-C_1_ structure. The most common derivatives of hydroxybenzoic acid found in plants are gallic, vanillic, *p*-hydroxybenzoic, syringic, and protocatechuic acids [33]. Hydroxycinnamic acids, which were widely distributed in plant-originated food products, are a class of polyphenols with a C_6_-C_3_ skeleton, and exist either in free form or associated with other components, such as quinic acid or polysaccharides [35,36]. Furthermore, volatile phenols possess aroma characteristics, and stilbenes can act as phytoalexins, which also contribute to flavor and potent biological activities for plants, respectively.

**Figure 2:**
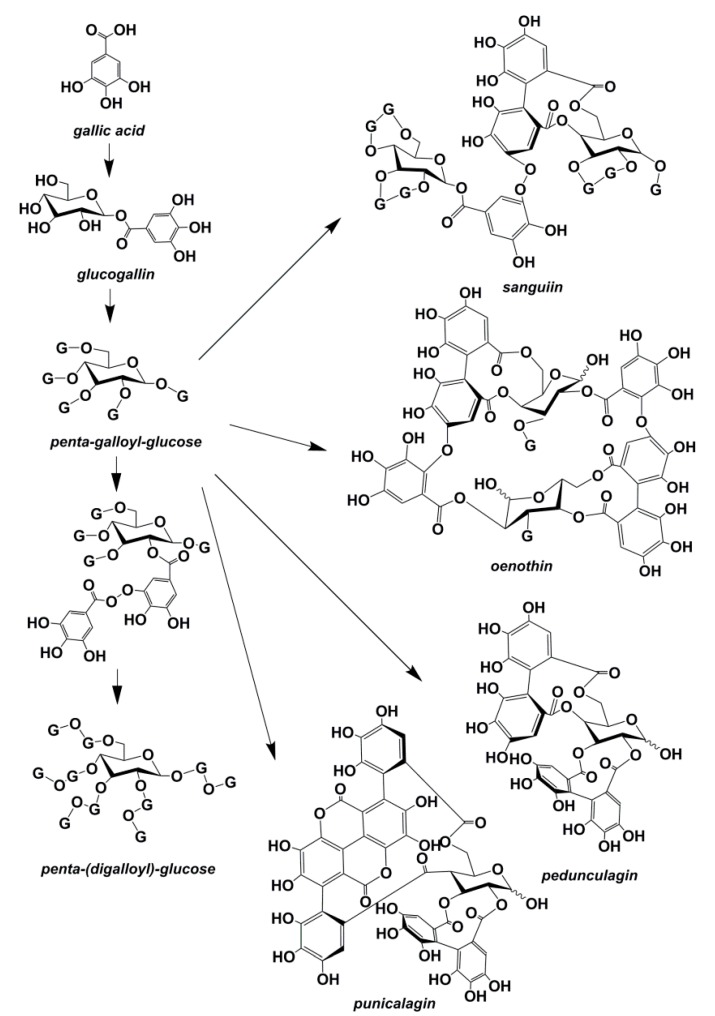
Structures of hydrolyzable tannins [39].

Unlike the classes of plant polyphenolics described above, tannin is a term applied to a large group of polyphenolics, which have a wide range of structures and are of intermediate to high molecular weight. Plant tannins are conventionally classified into two major classes: condensed and hydrolyzable tannins [28]. Condensed tannins, also known as proanthocyanidins, are widespread in all ferns, gymnosperms, and some angiosperms, which have flavonoid cores as their main basic unit (see the structure in Table 2) [37]. Hydrolyzable tannins, such as gallotannins and ellagitannins are polymers of glucose esters of gallic acids and hexahydroxydiphenic acids respectively, with molecular masses ranging from 500 to 5000 Da, which are only encountered in 15 of the 40 orders of dicotyledons [38]. Figure 2 illustrates the basic structures of hydrolyzable tannins.

## 3. Oak Polyphenolics

Polyphenolic compounds found in oak wood have important functions in aged wines. Not only do oak polyphenols have an essential role in wine maturation but also feature as influencing factors associated with potent antioxidant effects [40]. These compounds are normally classified into three main classes: volatile phenols, phenolic acids and ellagitannins. In structure, both volatile phenols and phenolic acids are derived from the simple phenol structure of hydroxybenzene, which is an aromatic ring, whereas ellagitannins are a diverse class of hydrolyzable tannins, a type of polyphenol formed primarily from the oxidative linkage of galloyl groups in β-1,2,3,4,6-pentagalloyl glucose.

### 3.1. Volatile Phenols

Volatile phenols are simple phenols in oak woods that can be extracted into wines, modifying their aroma compound profiles. Traces 4-vinylphenol was found in oak, but its content could approach the olfactory thresholds (605 μg/L) in wines aged over a long period of time, causing aromas described as “stable” and “medicinal” [41]. 4-Ethylphenol, another phenol derivative found in oak-aged wines, had horsey, leather, and sweaty saddle-like descriptors when its concentration was above 425 μg/L [42]. In contrast, guaiacol and its derivatives possess lower olfactory thresholds. Guaiacol and 4-methylguaiacol, which have smoky and spicy aromas have olfactory thresholds of 25 and 65 μg/L, respectively [43]. 4-Ethylguaicol with a 33 μg/L olfactory threshold also contributes spicy, toasted, and smoky aromas. Vinylguaiacol, with an olfactory detection threshold of 40 μg/L, contributes spicy, clove-like, and oak aromas [43,44].

**Figure 3:**
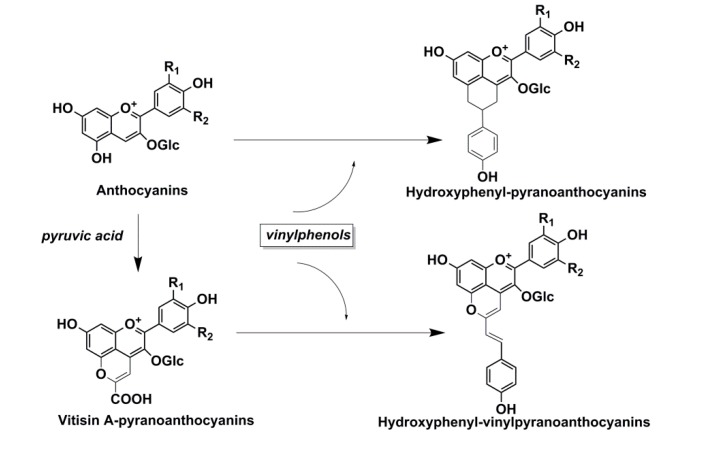
Formation of the anthocyanin-vinylphenol adducts [46].

Some studies showed that ethylphenol, vinylphenol and vinylguaiacol were present at low concentrations in oak wood. However, the amounts found in wines are mainly from the action of microorganisms. Microorganisms in the barrel, especially lactic bacteria and yeasts, are associated with these off flavors [45], such as ethylphenol, which its occurrence is normally attributed to the action of *Brettanomyces* yeast rather than oak directly. On the other hand, 4-vinylphenol and 4-vinylguaiacol could adduct with anthocyanins at their C_4_ and C_5_ positions and then undergo an oxidation process, incorporated into the pyran ring in the resulting pyranoanthocyanins. Such addition compounds could change the anthocyanin color to orange and protect the anthocyanins from hydration and so stabilize the anthocyanin pigments [46]. Figure 3 presents the formation of such an anthocyanidin-3-*O*-glucoside-vinylphenol adduct.

### 3.2. Phenolic Acid

**Figure 4:**
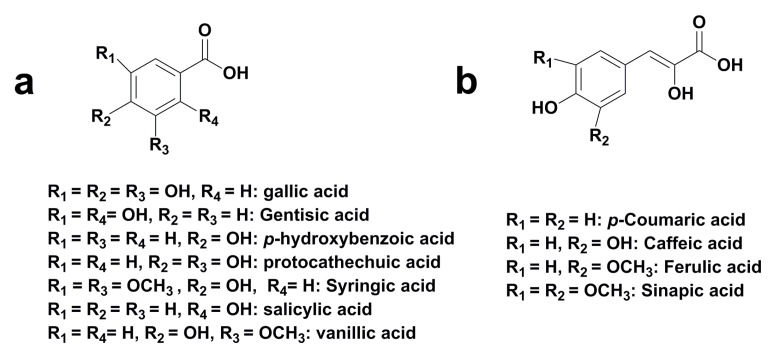
Chemical structures of phenolic acids in oak wood [47,48,49].

The main phenolic acids compounds identified in some seasoned and toasted oak are hydroxybenzoic and hydroxycinnamic acids [10]. Hydroxybenzoic acids which are derived directly from benzoic acid, include gallic, gentisic, *p*-hydroxybenzoic, protocatechuic, syringic, salicylic, and vanillic acids (Figure 4a). Related compounds also include hydroxybenzoic aldehydes, such as syringaldehyde and vanillin, which are modified as aldehydes with carboxyl groups. Hydroxycinnamic acids and their derivatives had the C_6_-C_3_ structures. The most common hydroxycinnamic acids in oak woods are *p*-coumaric, caffeic, ferulic acids, and sinapic acid (Figure 4b). Table 3 lists phenolic acids that have been reported in literatures.

**Table 3:** Phenolic acids in oak woods.

Compounds	Sample	λ (Max)	*M*_W_	ESI-MS/MS Prominent Ions (*m*/*z*)	References
**Hydroxybenzoic Acids**					
Gallic acid	A, B, D–I	270	170	169 [M-H]^−^, 154, 125, 81, 79	[5,40,50,51,52,53,54,55,56]
Gentisic acid	B	340	154	153 [M-H]^−^, 109	[50]
*p*-Hydroxybenzoic acid	H	255	138	137 [M-H]^−^, 93	[55]
Protocatechuic acid	A, B, H	259, 293	154	153 [M-H]^−^, 109	[40,50,55]
Syringic acid	B, D–I	275	198	197 [M-H]^−^, 182, 167, 153, 138	[5,50,51,52,53,54,55]
Vanillic acid	A, B, D–G, I	260, 291	168	167 [M-H]^−^, 152, 123, 108	[40,50]
*p*-Hydroxybenzoic aldehyde	B	280, 254	122	121 [M-H]^−^	[50]
Protocatechuic aldehyde	A, B, H	280, 310	138	137 [M-H]^−^	[40,50]
Syringaldehyde	A, C–I	307	182	181 [M-H]^−^, 166, 151	[40,51,52,53,54,55,56,57]
Vanillin	A–I	304	152	151 [M-H]^−^, 136, 108	[5,40,50,51,52,53,54,55,56,57]
**Hydroxycinnamic Acids**					
*p*-Coumaric acid	A, B, G	310	164	163 [M-H]^−^, 119, 94	[40,50,54]
Caffeic acid	A, B	324	180	179 [M-H]^−^, 135	[40,50]
Ferulic acid	A, B, E, F	280, 340	194	193 [M-H]^−^, 178, 149, 134	[5,40,50,53]
Sinapic acid	A, B	340	222	221 [M-H]^−^, 164, 149, 121	[40,50]
Sinapaldehyde	A, D–F, I	254	208	207 [M-H]^−^, 192, 189	[5,40,51,52,53,56]
Coniferaldehyde	A, D–F, I	260	178	177 [M-H]^−^, 162, 159, 147	[5,40,51,52,53,56]
Eugenol	D, E, G	230	164	163 [M-H]^−^, 149, 137	[51,52,54]

A: Toasted and non-toasted oak wood samples (American, French, Hungarian, Rumanian and Russian oak woods); B: Oak aged wine samples (American, French and Hungarian oak woods aged over 12 months); C: Oak wood samples; D: Oak aged sugarcane spirit samples; E: Green oak samples (Spanish oak wood); F: Natural seasoning and tasted oak wood samples (American, French and Spanish oak woods); G: Oak aged sugarcane spirit samples (*Quercus* spp. oak wood aged over 12 months); H: Oak aged rum samples (Oak wood aged over 15 years); and I: Oak chips samples (American oak wood).

Some studies have found that the color of red wines to be strongly influenced by the presence of phenolic acids [58]. These acids had an important function to enhance and stabilize the pigment of red wines by intra- and intermolecular copigmentation reactions, especially the hydroxycinnamic acids could provide approximately 60% to 70% color enhancement at 520 nm [59,60]. Table 4 shows the copigmentation effects (bathochromic shift and hyperchromic effect) of the four hydroxycinnamic acids.

**Table 4:** The compigmentation effects of cyanidin-3-*O*-glucoside (Cy-3-glu) and cyanidin-3-*O*-sophoroside (Cy-3-soph) with four hydroxycinnamic acids [61].

Anthocyanins	pH	Hyperchromic Effect (%)				Bathochromic Shift (∆ λ _max_, nm)			
		Coumaric Acid	Caffeic Acid	Ferulic Acid	Sinapic Acid	Coumaric Acid	Caffeic Acid	Ferulic Acid	Sinapic Acid
Cy-3-glu	3.2	11.6	18.8	25.3	29.7	2.5	4.5	6.6	8.7
	4.0	27.7	41.0	63.9	110.5	2.2	4.0	6.8	10.2
Cy-3-soph	3.2	9.9	11.2	16.5	23.9	2.5	3.8	5.2	5.5
	4.0	16.3	22.7	40.7	60.9	2.3	2.6	6.5	7.7

### 3.3. Ellagitannins

**Figure 5:**
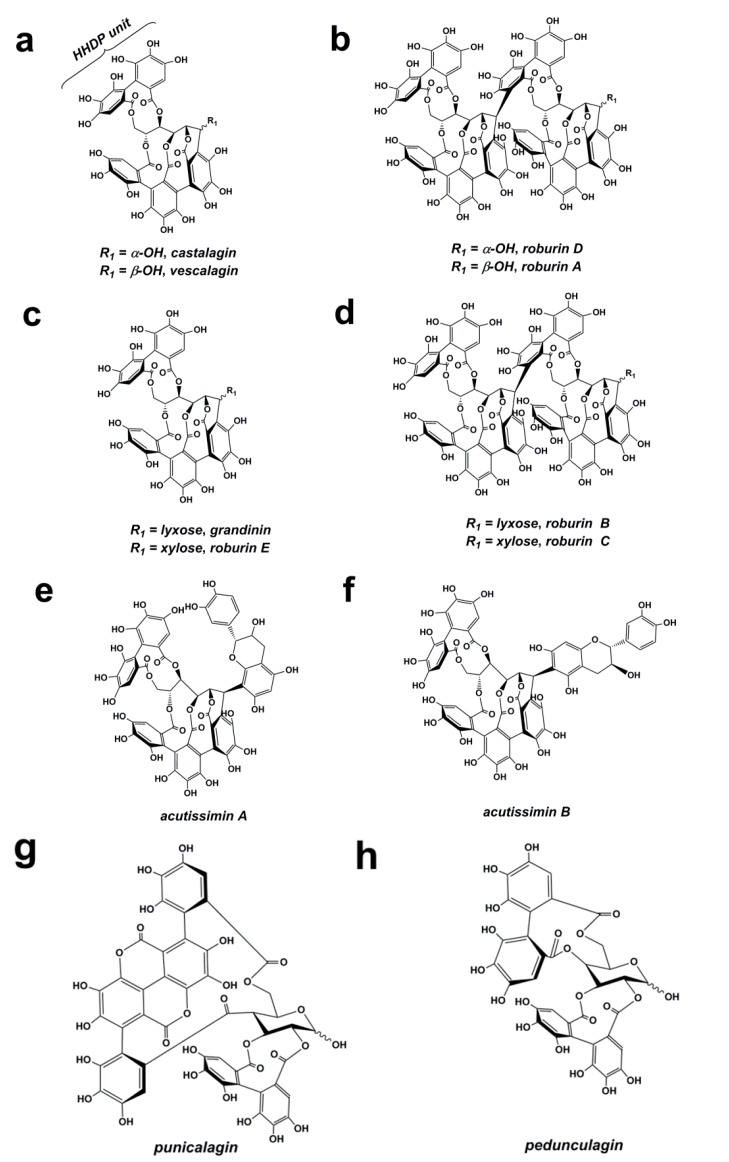
Chemical structures of ellagitannins [18].

Ellagitannins (Figure 5) constitute one of the major classes of polyhydroxyphenyl-bearing polyphenols derived from the secondary metabolism of plants. In oak, both sapwood and heartwood contained ellagitannins [62]. Their structures are characterized by one or more hexahydroxydiphenoyl (HHDP) moieties esterified with a sugar, typically d-glucopyranose [63]. HHDP moieties were further connected with neighboring galloyl residues through C-C biaryl ether bonds as a result of phenolic oxidative coupling processes [64,65]. Ellagitannin compounds have an enormous structural variability because of the different linkages of HHDP residues with the glucose moiety and their strong tendency to form dimeric and oligomeric derivatives [65]. Monomeric vescalagin and castalagin (Figure 5a) are normally found in oak species used in barrel making, which largely predominate in oak wood representing 40%–60% of the ellagitannins [18]. Dimers (roburin A and roburin D, Figure 5b) and lyxose/xylose derivatives (grandinin, roburin B, roburin C, and roburin E, Figure 5c,d) are also identified in oak woods [66]. In addition, flavanols, including dihydroflavonols, often occur in association with ellagitannin to form flavano-ellagitannin derivatives (such as acutissimin A and acutissimin B, Figure 5e,f), detected in aged wine and whisky, and gallagyl-glucosides, whose structure has an ellagic acid moiety (punicalagin, peduncalagin, Figure 5g,h), also belong to the same group [39]. The level of their occurrence in the cooperage wood depended partly on the species used, as well as the type and length of drying and toasting [67,68]. Many distinctive properties of oak could be attributed to these ellagitannins. For example, ellagitannins are toxic to microorganisms, and could prevent the rapid decay of the wood [69]. Therefore, the relative abundance of ellagitannins in oak wood endows oak with good resistance to fungal degradation. The combined presence of ellagitannins and tyloses in oak heartwood has also made these woods unrivaled in the manufacturing of barrels used in wine and whiskey aging [70]. Both acutissimin A and acutissimin B have been reported to possess interesting biological properties such as antitumoral to inhibit human DNA topoisomerase IIα *in vitro* [71]. Furthermore, ellagitannins could directly affect wine color. To the best of our knowledge, the reaction between ellagitannin and purple or red-colored grape-derived pigment could get furnished a novel orange-colored anthocyano-ellagitannin compound [72].

### 3.4. Polyphenolic Compounds in Non-Oak Woods

Although aging of wines were carried out solely and exclusively with oak wood, the dilemma of huge demand and limited resources required researchers to look for alternative species for coopering. In this process, the phenolic fraction was considered to be one of the most important parameter for evaluating quality in the choice of good alternative aging wood [73]. Research suggested that chestnut (*Castanea* *sativa*), acacia (*Robinia pseudoacacia*), cherry (*Prunus avium*), ash (*Fraxinus excelsior* and *F.* *vulgaris*) and mulberry (*Morus alba* and *M.* *nigra*) had been considered as possible sources of materials for cooperage to give a particular personality to aged wines and other derived products, such as cider, spirits and vinegar [74,75,76]. Some authors also highlighted that wines or vinegars aged in non-oak barrels had better organoleptic characteristics.

Chestnut wood was widely used for enological purposes in the Mediterranean region in the past. This was not only because they are especially rich in gallic acid and ellagitannins, but also because of their widespread availability and lower cost. Wines aged in chestnut wood contained high amounts of phenolic compounds, which could protect must from undesirable oxidation reactions, and stabilize the coloring compounds and improve the astringency of the wines [73]. Consequently, chestnut wood is the only alternative to oak that has been approved by the International Organization of Vine and Wine (OIV) for wine ageing. In recent years, there has been renewed interest in the phenolic composition of chestnut wood. Results revealed that its heartwood has the most similar polyphenolic profile to oak. However, there were also some differences. For example, 1-*O*-galloyl castalagin, which was for the first time found in chestnut samples, might originate from the esterification of castalagin or vescalagin with a gallic acid residue [77].

Some published work on the chemical composition of *R. pseudoacacia* wood, commonly known as false acacia, pointed out important chemical differences in relation to the oak wood that needs to be taken into account when considering that for cooperage [74]. Data in the literature showed that the acacia heartwood contained high amounts of flavonoids, dihydrorobinetin and robinetin, at the concentrations of up to 100 μmol/g. These compounds are characteristic markers of acacia wood, since they have not been detected in other woods used for cooperage, such as oak, chestnut, cherry and mulberry [76]. In contrast to those sources, acacia has been found to have only a small amount of condensed tannins and no hydrolysable tannins. Moreover, the low molecular weight phenolic compounds with a β-resorcylic structure in both acid and aldehydic forms were different to other woods [74,76].

Cherry wood is abundant in condensed tannins (procyanidin type). Additionally, some phenolic acids and their esterification products such as benzoic acid, *p*-hydroxybenzoic acid, 3,4,5-trimethylphenol, *p*-coumaric acid, methylsyringate and methylvanillate, and flavonoids such as naringenin, aromadendrin, isosakuranetin and taxifolin, could be used as its phenolic markers for authenticity purposes [75,78]. For ash wood, the presence of secoiridoids, phenylethanoid glycosides, or *di-* and oligolignols could be used as good identifiers [78]. According to the previous reports, mulberry could be identified by its high levels of polyphenols, in particular oxyresveratrol and its glycosides, as well as coumarin glycosides. However, although the different phenolic compounds can be used as characteristic markers to distinguish the type of wood, the polyphenolic profile variability of beverages such as wine and oak aged spirits, and their evolution during ageing, makes the analysis of the markers found in the wood more complex than for the woods themselves [79].

## 4. Some Factors Affecting the Polyphenolic Profile of Oak Woods

The concentration of polyphenolics extracted from wood into wines during aging mainly depends on the pool of these compounds present in the barrels’ wood, as well as the aging conditions. Towey and Waterhouse [80] stated that the structures and amounts of polyphenolics could be affected by the species of the wood and other factors, including geographical origin [70,81,82,83], silvocultural treatment, drying treatment, degree of toasting [81,84], and the length of time wines spent in the barrels. In addition, barrel age and usage (*i.e.*, the number of times a barrel was used) [85], and barrel volume also affected the polyphenol contents in wines.

### 4.1. Oak Species and Geographical Origins

In the barrel industry, three wood sources are most frequently used: American white oak (*Q. alba*) and two French red species (*Q. petraea* and *Q. robur*). The major differences between these oak woods are as follows. American oak wood is characterized by a lower total ellagitannin and significantly higher quantities of vanillin content than the French oak. However, *Q. robur* had higher amounts of gallic, protocatechuic, caffeic, sinapic acids, and total phenolics compared with *Q. alba* [7,86]. Nonetheless, pedunculate (*Q. robur*) and sessile (*Q. petraea*) oaks were not significantly different in their ellagitannin content [7,86,87]. During oak harvest, French oak samples, from the Troncais and Vosges regions, were found to contain about twice of eugenol of American oak samples. However, after three years of open-air seasoning, the eugenol concentration of French oak samples decreased by 60% to 70%, whereas the decrease in the American oak samples was minimal [88]. Interestingly, even within a single species, there was high variability in polyphenolic composition that has been recognized in French oak from different geographic regions. Oak wood from the southwest (e.g., Limousin), center (e.g., Troncais, Nevers, and Allier), and northeast (e.g., Vosges) of France could also be differentiated based on their differing vanillin and eugenol contents [89,90]. Prida and Puech studied 56 American oaks, 276 French oaks, and 102 Eastern European oak trees and found that ellagitannins were the best substances for distinguishing the species, whereas eugenol, 2-phenylethanol, and the phenolic aldehydes vanillin and syringaldehyde, which had higher concentrations in oak samples from Eastern Europe, were the best components to be used as variables for distinguishing geographic origins [91]. Several studies on the polyphenolic composition of Spanish oaks indicated that low molecular weight phenolic and tannic profiles of Spanish species were similar to those of French oak, and quantitative differences often decreased with seasoning and toasting processes [8,53]. Phenolic parameters and sensory analysis showed that wines aged with the Spanish *Q. pyrenaica* had enological characteristics similar to those aged in American or French oak wood using barrels, chips, or staves [22,92]. Researchers thus concluded that Spanish oak wood was a potential alternative to traditional oak species based on their phenolic compounds.

### 4.2. Cooperage Treatment

During barrel production, the oak wood used in cooperage undergoes several processing steps which affect the composition of the wood. Seasoning and toasting processes especially critically affected the polyphenol composition [81,84].

#### 4.2.1. Seasoning

The natural seasoning of oak is more than a simple dehydration process. There is a loss of water soluble polyphenolic substances, which contributes to a decrease in the sensations of bitterness and astringency in wines [93]. During the seasoning treatment, the concentrations of different ellagitannins decreased according to the duration of this process, and this loss was predominant in the first few millimeters of each stave face over the first three years [8]. This decline was thought to be partly due to hydrolysis reactions. In contrast drying through outdoor, seasoning of oak increased the levels of volatile phenols and phenolic aldehydes, including eugenol, vanillin, syringaldehyde, coniferaldehyde, and sinapaldehyde, especially when seasoning was carried out in hot climates [50]. Simón *et al.* [94] analyzed four oak wood samples in Spain and concluded that the concentrations of polyphenolic compounds differed according to the seasoning time and species. Their research also showed that ellagic acid was the main component in unseasoned wood, followed by gallic acid. With respect to the remaining components, aldehyde levels were always higher than those of acids, and cinnamic compounds were more abundant than benzoic acids, except for ferulic acid, which was present at very low concentrations [84].

#### 4.2.2. Toasting

Toasting, the next step of cooperage treatment, induces severe chemical modification to a range of compounds in the wood. The level of toasting is generally referred to in terms of light (toasting for 5 min to reach a temperature of 180 °C), medium (toasting for 35 min to reach a temperature on the wood surface of 160–170 °C), and heavy (toasting for 17.5 min to reach a temperature of 230 °C). During this process, a variety of hydrothermolysis and pyrolysis reactions takes place depending on the degree of toasting. Pyrolysis and hydrothermolysis degraded wood constituents to some extent, including not only the ellagitannins, which are readily hydrolyzed [8,95,96], but also the lignin and hemicelluloses, which are subsequently altered. Lignin was a tridimensional polymer made up of two phenylpropanoid alcohols, namely, 4-hydroxy-3-methoxycinnamic alcohol (coniferyl alcohol) and 4-hydroxy-3,4-dimethoxycinnamic alcohol (syringyl alcohol). In toasted wood, a variety of mono- and dimethoxylated phenols and high levels of cinnamic acids, benzoic acids, and aldehydes were formed from these two alcohols by hydrolytic and oxidative processes. The degradation of these compounds would contribute to increase the levels of phenolic aldehydes and other components in the wood with desirable sensory effects. In contrast, non-toasted oak wood only had a small quantity of volatile phenols, mainly eugenol, and traces of phenolic aldehydes [97,98]. Toasting also had an important influence on the ellagitannin compositions of the oak. Roburins A–E, grandinin, vescalagin, and castalagin decreased during this process [8]. An increase in the ellagic acid content of toasted samples was also observed, which might be due to the release of the compound by ellagitannins during thermal degradation [40]. French coopers had, perhaps, used toasting to lower the tannin level, thereby limiting the increase in a wine’s astringency. Doussot *et al.* [68] found that medium toasting drastically enhanced the loss of ellagitannins (>90%) but increased volatile phenols by 30%. On the other hand, other phenolic compounds, such as gallic, protocatechuic, caffeic acids, and scopoletin, were also sensitive to thermal degradation, causing a significant decrease in their content in the toasted wood [99].

#### 4.2.3. Aging Process and Barrel Properties

A series of chemical reactions occurs during barrel aging of wines. The length of time that a wine spends in a barrel and the barrel volume affects the polyphenolic content in wines. Sanza *et al.* [50] evaluated the aged wines treated under different systems (barrels, oak chips, and oak staves) and found an increase in the concentration of *p*-coumaric, ferulic, gallic, protocatechuic, caffeic acids, and protocatechuic aldehyde during aging. Several beverages, including cognac, whiskey, brandy, vinegar, mescal, and wine increased their antioxidant capacity as a result of wood contact [22]. This was due to the significant amount of polyphenols extracted by these beverages from the wood during aging. Therefore, the nature and content of polyphenolics of the wood used in aging could determine the increase in antioxidant capacity of wines during storage [100].

Once oak components were extracted out of wood, they underwent a series of micro-oxygenation alterations. Specifically, phenolic aldehydes could change to their respective acids with subsequent ethyl ester formation. Some winemakers also recommended that micro-oxygenation could be used as a complementary technique to oak aging when oak barrel alternatives were used. Pérez-Prieto *et al.* [101] studied wines matured in oak barrels of different capacities (220, 500 and 1000 L) and found that guaiacol and 4-methylguaiacol was extracted rapidly during the first several days of oak maturation. In contrast, vanillin required at least 3 months to accumulate in wines. However, contrary to these results, Spillman *et al.* [102] found that the rate of vanillin extraction was very fast during the first two weeks of oak storage in a hydroalcoholic medium. The volatile phenols 4-ethylphenol and 4-ethylguaiacol were formed in large quantities after the first 90 days of oak maturation. These findings were in agreement with the results obtained by Pérez-Prieto *et al.* [101]. Wines matured in 1000 L oak barrels accumulated the least amounts of volatile compounds. Rodríguez-Rodríguez *et al.* [103] also compared the wines in different volumes (300 and 500 L) of French oak barrels and found that wines aged in the smaller barrel with a medium toast level acquire better chromatic characteristics, higher amounts of oak-related aroma compounds, and higher sensory analysis scores.

#### 4.2.4. Others

Other factors might also affect the polyphenol contents of oak, such as the soil type, sun exposure, and rainfall where the oak trees were cultivated. Many authors have reported a decline in soluble ellagitannins with increasing heartwood age [104]. This decline was believed to be partly due to hydrolysis reactions and mostly due to non-enzymatic oxidative polymerization reactions, which might reduce tannin solubility [105,106]. Mosedale *et al.* [107] found no difference in the heartwood ellagitannin content when clonal and progeny trials of oaks (*Q. petraea* and *Q. robur*) were analyzed.

## 5. Analysis of Polyphenolics in Oak Woods

With the advent of the modern scientific revolution and the development of chemistry from alchemy, plant polyphenols became some of the first subjects of scientific researches. Many research worked in this field and provided the first results in relation to the quantitative and qualitative analyses of polyphenolic compounds [39]. Given the large number and the chemical complexity of these polyphenolics, considerable progress in terms of extraction methods and analytical techniques have been made, especially over the last few decades. Different analytical methods, such as stir bar sorptive extraction, solid phase microextraction (SPME), and high performance liquid chromatography (HPLC), have been studied and described. The characterization of polyphenolic compounds relied on the suitability of these methods for the analysis of plant tissues. The next section summarizes the analytical chemistry applications and tools currently used to analyze polyphenolics in oak wood, and other sources, with similar polyphenolic composition. Table 5 lists representative examples of the methods used for separation of (poly)phenols.

**Table 5:** Overview on methods for analysis of (poly)phenolics from oak woods and other different sources.

Source		Sample Preparation	Analytical Techniques	Main Polyphenolic Compounds	References
Oak heartwood (2006)		Extracted sawdust with methanol/water (1:1); Filtration and evaporated; Residue extracted in diethyl ether and ethyl acetate	HPLC-DAD	Gallic acid, vanillic acid, vanillin, syringic acid, syringaldehyde, ellagic acid, coniferylaldehyde, sinapic aldehyde, scopoletin, castalagin, vescalagin, grandinin, roburin A–E	[52]
Oak and Brazilian woods (2009)		Extracted sawdust with sugarcane ethanol (47% *v*/*v*), followed by separation using C18 Sep-Pak	HPLC-DAD-fluorescence and HPLC-ESI-MS	(+)-Catechin, coniferaldehyde, coumarin, ellagic acid, (−)-epicatechin, eugenol, gallic acid, myricetin, quercetin, scopoletin, synapaldehyde, syringaldehyde, syringic acid, *trans*-resveratrol, vanillic acid, vanillin	[51]
Oak heartwood (2011)		Extracted sawdust with methanol/water (1:1); Filtration and evaporated; Residue extracted in diethyl ether and ethyl acetate	HPLC-DAD	Gallic acids, ellagic acids, vanillic acids, syringic acids, ferulic acids, vanillin, syringaldehyde, coniferaldehyde, sinapaldehyde, aesculetin, scopoletin	[53]
Oak heartwood (2011)		Extracted sawdust with methanol/water (1:1); Filtrated and evaporated; Residue extracted in diethyl ether and ethyl acetate	HPLC	Roburins A–E, grandinin, vescalagin, castalagin	[8]
Cherry heartwood (2010)		Extracted sawdust with methanol/water (1:1); Filtrated and evaporated; Residue extracted in diethyl ether and ethyl acetate	HPLC-DAD/ESI-MS	Gallic acid, protocatechuic acid, vanillic acid, vanillin, syringic acid, syringaldehyde, 3,4,5-trimethoxyphenol, protocatechualdehyde, benzoic acid, *p*-hydroxybenzoic acid, *p*-coumaric acid, scopoletin, coniferaldehyde, coniferaldehyde, methyl vanillate, methyl syringate	[108]
Black pine bark (2010)		Extracted with pressurized hot water; Filtrated; Washed with chloroform, ethyl ether and other solvents	HPLC, CC, TLC and FT-NMR	(+)-Catechin, (−)-epicatechin, quercetin, ferulic acid	[109]
Acacia heartwood (2011)		Extracted sawdust with methanol/water (1:1); Followed by extraction with ethyl acetate	HPLC-DAD and LC-DAD/ESI-MS/MS	Gallic acid, gallic aldehyde, protocatechualdehyde, methyl gallate, β-resorcilyc acid, vanillic acid, β-resorcilyc aldehyde, caffeic acid, vanillin, syringic acid, syringaldehyde, coniferaldehyde, sinapaldehyde, ellagic acid, robtin *etc.*	[110]
Red wine (2008)		Solid phase extraction	HPLC-UV	Monomeric, oligomeric and polymeric polyphenolic compounds	[111]
Red wine vinegar produced in barrels made from different woods (2008)		Direct injected	HPLC-UV	Gallic acid, protocatechuic acid, tyrosol, caftaric acid, vanillic acid, (+)-catechin, caffeic acid, syringic acid, (−)-epicatechin, resveratrol glucoside, ellagic acid	[112]
Vinegar (2012)		Extraction of vinegar with stir bar at 1250 rpm, 25 °C for 120 min	GC-MS	Benzaldehyde, benzaldehyde, phenol, 4-acetyl-2-methylphenol, 4-acetyl-2-methylphenol, *p*-ethylguaiacol, 4-ethylphenol, 2,4-ditertbutylphenol, benzoic acid	[113]
Pomegranate beverage (2012)		Direct injection	HPLC-DAD-MS	Gallic acid, (+)-catechin, (−)-epicatechin, caftaric acid, ellagic acid, myricetin, quercetin, *etc.*	[114]
Apple pomace (2009)		Extracted apple pomace powder with ethanol; Concentrated and vacuum dried; fractionated polyphenols using Sephadex LH-20; HSCCC separation	HPLC-MS	Chlorogenic acid, quercetin-3-glucoside, phloridzin, quercetin-3-glacaside, quercetin-3-xyloside, quercetin-3-arabinoside and quercetin-3-rhamnoside	[115]
Apple pomace (2010)		Extracted with ethanol and assisted by microwave treatment	HPLC-UV	Chlorogenic acid, cafeic acid, syrigin, (−)-epicatechin, procyanidin B2, cinnamic acid, coumaric acid, phlorizin, quercetin	[116]
Onion (2008)		Extract with 80% methanol; Filtrated	HPLC-UV	Kaempferol, quercetin, isoquercetin, quercetin monoglucoside, quercetin diglucoside	[117]
Onion (2009)	Extracted with methanol:formic acid:water (50:5:45)		HPLC-DAD	Quercetin-3,4'-diglucoside, quercetin-4'-glucoside, cyanidin-3-glucoside, cyanidin-3-laminaribioside, cyanidin-3-(6''-malonyl-glucoside), cyanidin-3-(6''-malonyl-laminaribioside)	[118]
Onion (2010)	Extracted with methanol:formic acid:water (50:5:45)		HPLC-DAD	Quercetin-3-glucoside, quercetin-3,4'-diglucoside, quercetin-4'-glucoside, quercetin-7,4-diglucoside, isorhamnetin-4-glucoside, isorhamnetin-3,4-diglucoside, cyanidin-3-glucoside, cyanidin-3-laminaribioside, cyanidin-3-(6''-malonyl-glucoside), cyanidin-3-(6''-malonyl-laminaribioside)	[119]
Roasted wheat germ (2009)	Supercritical carbon dioxide extraction		HPLC-MS	Ferulic acid, vanillic acid	[120]
Pistachio (2010)	Extracted crushed seeds and skins with methanol/water (2:1); ultrasonicated; Homogenate centrifuged and separated		HPLC-DAD	Gallic acid, eriodictyol-7-*O*-glucoside, catechin, naringenin-7-*O*-neohesperidoside, quercetin-3-*O*-rutinoside, eriodictyol	[121]
Potato peels (2011)	Peels lyophilized and ground; Extracted with methanol assisted by microwave treatment		HPLC-UV	Chlorogenic acid, caffeic acid, ferulic acid, rutin	[122]
Apple Seeds (2012)	Extraction of defatted apple seeds with aqueous acetone (30:70; *v*/*v*)		HPLC-DAD	Phenolic acids, chlorogenic acid, phloridzin, phloretin-2'-xyloglucoside, flavan-3-ols, quercetin-3-*O*-glucoside	[123]
Rooibos (2011)	Extraction with distilled water; Followed by filtration and re-extraction with ethanol (80%; *v*/*v*)		HPLC-ESI-MS	Esculin, rutin, quercetin, isoquercitrin, luteolin, nothofagin, secoisolariciresinol, *etc.*	[124]
Rooibos (2012)	Extraction with boiled deionized water; Then filtrated		HPLC-DAD	Phenylpyruvic acid-2-*O*-glucoside, isoorientin, orientin, aspalathin, ferulic acid, quercetin-3-*O*-robinobioside, vitexin, hyperoside, rutin, isovitexin, isoquercitrin, nothofagin	[125]
Mate tea (2012)	Extraction of leaves with ethanol/water; Followed by filtration		HPLC-DAD	Chlorogenic acid	[126]
Grapes (*V. vinifera* L.) (2012)	SPME		GC-MS	Phenol, 2-methylphenol, eugenol, 2-methoxy-4-vinylphenol	[127]
Mango (2012)	Extraction with 80% methanol and 2% formic acid; Followed by extraction with 80% methanol		HPLC-DAD-MS/MS-ESI	Gallic acid, protocatechuic acid, chlorogenic acid, vanillic acid	[128]
Olive oil (2012)	Extraction with *n*-hexane and assisted with an ultrasonic probe; Centrifugation and separation; Followed by extraction with methanol		HPLC-DAD-FLD and LC-MS	Hydroxytyrosol, tyrosol, oleuropein aglycone derivative and ligstroside derivative	[129]

### 5.1. Extraction

The range of phenolic compounds extracted from plant materials is influenced by the chemical nature of the compounds, the extraction method used, the sample particle size, the extraction time and conditions, and the presence of interfering substances. Phenolic extracts of plant materials are composed of a mixture of different classes of phenolics determined by their solubility in the solvent system used [130,131]. Although the plant matrices are various, surprising similarity in the extraction systems could be observed in extracting polyphenols and their derivatives. Methanol, ethanol, acetone, water, ethyl acetate, and, to a lesser extent, propanol, dimethylformamide, and their combinations were frequently used to extract polyphenolics [132]. Non-polar organic solvents (*i.e.*, *n*-hexane, petroleum ether, chloroform, and dichloromethane) have relatively low extraction abilities for polyphenols, and were usually used in sample treatment to remove lipids and chlorophyll and/or to prevent enzymatic reactions [41]. Moreover, given that some extracted phenols were present as glycosides, caution was exercised in extracting them to avoid hydrolysis, since alkaline hydrolysis might lead to significant losses of hydroxycinnamic acid derivatives [133]. Precautionary techniques, including isolation in the dark and under cold conditions were performed. Some extraction methods also used small amounts of ascorbic acid to avoid the polyphenol oxidase reaction and protect degradation of phenolic compounds [134,135].

Soluble phenolic compounds can be easily isolated from plant tissues by extraction with methanol or methanol acidified with 0.1% (*v*/*v*) HCl. Methanol/water (1:1) for example has been widely used to extract low molecular weight phenolic compounds (hydroxybenzoic and hydroxycinnamic acids) in oak wood at room temperature for 24 h [5], and absolute methanol/HCl mixtures was used to extract ellagitannins. However the latter solvent was more effective than the simple methanol/water solution [50]. In addition, the water and organic solvents used individually or in mixture, such as acetone/water/acetic acid (90/9.5/0.5) and ethyl acetate/methanol/water (60/30/10), significantly affected the total polyphenol contents extracted from *Q. coccifera* [136].

Besides the traditional solvent reflux extraction, supercritical fluid extraction, ultrasound extraction, and microwave extraction were favored alternative extraction methods compared with the first method because of their high efficiency and low solvent consumption. In some studies, the authors described an increase in total polyphenols by ultrasound [137,138]. This observed increase was attributed to the phenomenon of cavitation produced in the solvent by the passage of an ultrasonic wave, which accelerated the transfer of organic substances from plant materials. SPME, a relatively new extraction method, was another alternative to the aforementioned techniques because it was simple, rapid, and inexpensive. This technique does not require solvents, thereby reducing sample manipulation, and is easy to automate [139]. However, SPME was only used to determine a wide variety of volatile phenolic compounds with low molecular weight in oak products or oak-aged wines. For example, Díaz-Maroto *et al.* [140] used a Head Space-SPME (HS-SPME) method to rapidly extract volatile compounds (e.g., eugenol, vanillin, and syringaldehyde) from wines present in non-toasted and toasted oak wood of different origins.

In addition, with the development of science and technology, some new technologies and new methods have been applied to extract the polyphenolic compounds. Kadim *et al.* studied the mechanism and the kinetics of phenolic extraction from woods to wines during aging in barrels and found that the mass transfer rate was controlled by the rate of liquid penetration into the wood, rather than by diffusional transport of the phenolics [141]. Based on this finding, they developed a mathematical model to represent this migration, which satisfactorily predicted the shape of the extraction curve in the case of white wine aged in new barrels.

### 5.2. Separation

Phenolic extracts, first concentrated under vacuum, could be further extracted with petroleum ether, ethyl acetate, or diethyl ether to remove lipids and other unwanted foreign substances. Then, a variety of separation and identification methods could be used to characterize the phenolic compounds in them. The frequently used methods for separation and purification of polyphenols were various chromatographic methods, including paper chromatography (PC), thin layer chromatography (TLC), column chromatography (CC), HPLC, countercurrent chromatography (CCC) and some further developed methods.

PC, as an old analytical method that separated and identified mixtures, used to be very popular in the 1950s. During that time, many publications attested to the utility of this technique in the analysis of phenolic substances. For example, Bate-Smith separated anthocyanins, flavones, and other polyphenolic substances by using a butanol/acetic acid/water system [142]. Evaris *et al.* used butane/pyridine/aqueous sodium chloride and diazotized sulfanilic acid to separate and detect simple phenols and their derived phenolic acids [142]. Lindstedt applied PC to the separation and identification of nine phenolic constituents of pine heartwood extracts and found that the best results could be obtained with a water-saturated mixture of benzene and ligroin containing traces of methanol [143]. Quinn and Singleton [144] used 2D PC to analyze aqueous ethanolic extracts of three sources of oak woods. Ellagitannins have been identified in all oak wood extracts by using paper chromatographic *Rf* values. Although the PC method has largely been replaced by TLC, it is still a powerful tool.

TLC was also another chromatographic technique used to separate the mixtures. Generally, it was performed on a sheet of glass, plastic, or aluminum foil, which was coated with a thin layer of adsorbent material, usually silica gel, aluminum oxide, or cellulose (blotting paper) [145]. Lepri *et al.* [146] described the application of TLC to polyhydroxybenzenes and dichloro-, trichloro-, dinitro- and alkylphenols by using water/alcohol mixtures and silanized silica gel as the stationary phase. The polarity and strength, and hence pH, of the solvents used in the mobile phase could be varied depending on the separation method to obtain the desired effect. For instance, it was possible by varying the strength of the solvent system, such as 5% acetic acid or pure water, to better separate *di*- and *tri*-glycosides of flavonoids. In contrast, aglycones and monoglycosides were better resolved in 40% to 50% acetic acid. Once an appropriate separation method was applied and the stationary phase was dry, the separated compounds could be further analyzed by using visible light (chalcones) or UV (flavonoids, phenols, and hydrolyzable tannins). When phenolics after spray reagent treatment, were examined under daylight or UV, the colors could be used to identify their classes, since phenols usually showed blue, green, brown, and red colors, whereas flavonoids usually showed orange, yellow, and green hues. Some preliminary structural elucidation of phenolics could also be determined based on their fluorescent color under UV. Flavonoids, for example, range from dark purple (5,4'-OH flavones/flavonols with no free 3,3'- or 5'-OH) to dull yellow (flavonol aglycones) [147]. However, the yield from TLC plates was small, thus it was often used to analyze and identify samples, but not to separate them for the preparation of samples.

HPLC could be used to separate polyphenols in a liquid mixture. It was worth noting that this method was not bound by sample volatility or thermal stability, and could be conducted at room temperature, was easy to use, and generated powerful data. HPLC separation of phenolics could be classified into two groups: (1) Non-polar phenolics (separating well on silica columns with an isocratic elution); and (2) Polar phenolics (separating well on reverse-phase chemically bonded silica columns with gradient elution). Ordinarily, monomeric phenolics were most often identified and characterized by using reverse-phase HPLC, using a gradient solvent system with at least one acidic solvent to elute the compounds according to their ionic properties [148]. In grapes and wines, where some phenolic compounds are often found in highly polymerized forms, acid-induced depolymerization in the presence of a nucleophile, such as thiol or phloroglucinol, must be performed first. The subunit profiles and the mean degree of polymerization of the sample could then be determined [149]. Normal phase HPLC was used to estimate the size of polymeric phenols of up to seven units in length. The method developed by Kennedy and Waterhouse allowed the determination of low- and high-molecular weight polymers, as well as low- and high-molecular weight colored polymers or pigments [150]. However, this method was not commonly used during sample preparation because of equipment constraints and low yield. Altogether, HPLC is presently the most widely used quantification method [151,152,153].

CC was often used for the separation and purification of natural phenols. Alumina was previously the main packing material of CC, whereas resins and gels are more normally used today. Hathway used Solka Floc cellulose columns (60 cm × 5 cm) for the fractionation of tannins from oak bark pulps. An aqueous solution (125 mL) containing water-soluble phenolics was applied to the top of the column, from which the sugars and mobile phenolics were eluted with 5 L of 10% (*v*/*v*) formic acid [154]. Oak bark phlobatannin occupied a zone stretching from 2.5 to 7.5 cm from the top of the column. Better results might be obtained by vinyl polymer gels, such as Toyopearl and Diaion [14,155], and hydroxypro-pylated dextran gel (*i.e.*, Sephadex LH-20) [41]. Vidal *et al.* used a Toyopearl (TSK HW-50 column, 50 cm × 25 cm) equilibrated with water for a semi-preparative separation of anthocyanins extracted from grape skin with ethanol-water (75:25, *v*/*v*) containing 2% acetic acid. The condensed tannins are commonly separated by using Sephadex LH-20 CC. The crude extract was applied to the column and washed with ethanol to elute the non-tannin substances. Then, condensed tannins were eluted with acetone-water or alcohol-water [130,156].

CCC was recently explored as an alternative to liquid chromatographic techniques for the fractionation of various classes of phenolic compounds [130]. It uses an automated version of liquid-liquid separation, comparable with the repeated partitioning of an analyte between two immiscible phases by vigorous mixing in a separatory funnel. This technique is normally referred to as high-speed CCC (HSCCC). Regalado *et al.* used HSCCC for the preparative isolation of phenolic compounds in aged rum with a coil speed of 900 rpm. Solvent systems of *n*-hexane/ethyl acetate/methanol/water with 0.1% trifiuoroacetic acid (1:1:1:1 (*v*/*v*/*v*/*v*)) were used at a flow rate of 3 mL/min for the elution of phenolics in ascendant and descendant modes [55].

### 5.3. Identification

Almost every established method for polyphenolic identification usually has its limitations. The gold standard for structural identification of any organic compound was NMR spectroscopy [157]. This powerful technique allowed the structures of unknown molecules to be deduced with little prior information required. For example, Sudjaroen *et al.* used methanol as an extraction reagent to isolate polyphenolic components from Longan (*Dimocarpus longan* Lour) seeds followed by by ^1^H and ^13^C NMR for identification. The technique successfully identified five main compounds, including ellagitannin corilagin, chebulagic acid, ellagic acid 4-*O*-α-l-arabinofuranoside, isomallotinic acid, and geraniin [158]. Vivas *et al.* [159] applied NMR to elucidate that the two principal HHDP esters of ellagitannins in *Q. robur* heartwood were vescalagin and castalagin. The only limiting factor to NMR application was the requirement of relatively large amounts of the very pure samples, as well as its relatively high expense.

Mabry *et al.* introduced an alternative method based on UV-vis spectroscopy [160]. By obtaining spectra of hundreds of phenolics and their derivatives, many strong correlations were found between the chemical structures and their UV absorption characteristics. Generally, non-flavonoid and gallotannins had a characteristic peak at 280 nm, ellagitannins had a UV spectra below 270 nm, flavonoids, such as quercetin, absorbed maximally at 365 nm, and anthocyanins were easily distinguished by measurement at around 520 nm. For isomers, UV-vis spectral information alone could not be used for positive identification because co-eluting isomers could lead to spectra representing a mixture of the isomers. In this case, and for compounds with unavailable standards, additional means of identification should be used to interpret the separation, such as HPLC with a diode array detector (DAD) or mass spectrometry (MS) [28]. These methods are much simpler and easier than NMR, and the preliminary identification could be made without standards according to the literature. Besides, HPLC coupled with atmospheric pressure electrospray ionization (ESI)-ion trap mass spectrometry (ITMS) or triple quadrupole mass spectrometry (QQQ-MS) was one of the most widely used analytical techniques for targeted quantitative analysis of small molecules commonly found in analytic chemistry fluids. A less commonly used means of detection involves electrochemical and fluorescence detectors coupled to HPLC [142]. Cadahía and Simónet *et al.* determined the structures of several ellagitannins isolated from Spanish, French, and American oak woods by HPLC [8,52]. These compounds included roburins A–E, grandinin, vescalagin, and castalagin. The evolution of low molecular weight polyphenols in Spanish oak heartwood of *Q. robur*, *Q. petraea*, *Q. pyrenaica*, and *Q. faginea*, in relation to the processing of wood in barrel cooperage, was studied by HPLC [72]. In addition, the detection, using HPLC and MS in the positive mode, of flavonol glycosides in *Picea* and *Abies*s pecies, which belong to Pinaceae, have also been recorded [161]. About six 3-(6-acetyl-glucosides) and other acetyl-glucosides were identified. The most abundant sugar-moieties connected to the flavonoids were glucose and rutinose. Among them, the flavonol-rhamnosides were present only in the *Abies* species. However, these methods could only provide rapid access to compound molecular weight and sugar chain information. Spectral characteristics and mass information cannot precisely determine the structure of polyphenols. Combination with other means of identification, such as NMR, was still necessary to further explore the type and position of glycosidic bonds in polyphenolics.

## 6. Polyphenol Bioactive Ingredients in Other Plant Foods

Polyphenolic compounds are a group of biologically active molecules present extensively as metabolites in plants, which could be found in every part of fruits and vegetables mostly in the form of complex mixtures. Meanwhile, the varieties and contents of polyphenols in plants vary a lot depending on genotypes, species, environmental conditions, agronomic practices and the degree of maturity, *etc*. [162]. Some previous studies indicated that there were around 8000 kinds of polyphenols in plants while new polyphenols were discovered constantly. The characteristic phenols are various in different plants [163]. For instance, the primary polyphenols in strawberries (*Fragaria ananassa* Duch.), apples (*Malus pumila* Mill.), walnuts (*Juglans regia* L.), mangos (*Mangifera indica* L.), grapes (*Vitis vinifera* L.), and persimmons (*Diospyros kaki* L.) are respectively proanthocyanidins [164,165], phlorizin [166,167], juglone [168,169], mangiferin [170,171], resveratrols [172,173], and tannins [174,175]. These polyphenolic compounds in different plants could be used as the characteristic phenols to distinguish themselves or their nutritive values. For example, the astringency of persimmons is mainly caused by tannins; a large amount of lignins make the shell of the nuts hard; and resveratrols could be used as an indicator to identify the place of origin and raw materials of wine, as some species of grapes contain resveratrols.

With the improvement in people’ living standards and the enhancement in consumers’ health-care awareness, more and more research on the physiological functions of plant-derived polyphenols have received considerable attentions as potentially protective factors against cancer [176,177,178] and heart diseases [179] in part because of their potent antioxidative properties and their ubiquity in a wide range of commonly consumed foods of plant origin. Among other secondary plant metabolites, polyphenols are believed to contribute to the health protective effect of many food commodities. Therefore, they have even been named “vitamins of the 21st century” [180]. Yamanaka *et al.* reported that guava (*Psidium guajava* L.) leaf showed strong depression effect against *Vibrio* and *Aeromonas* species [181]. They analyzed the components of guava leaf drinks using HPLC-DAD and found that the drinks contained several kinds of tannins and polyphenolic compounds. After further separating the polyphenolic compounds, they discovered that ellagic acids, castalagin and casuarinin showed strong inhibitory effect against *Vibrio* and *Aeromonas*. As the major wintering fruits in the Mediterranean region, citrus fruits contain large amounts of flavonoids. Studies showed that they had high antioxidant activity which was of great help to replenish human body with antioxidant to fight against the damage caused by free radicals. Therefore, flavonoids become a major source of important nutrients for people living within this region [182]. In Finland, apples and onions are the main sources of dietary flavonoids that are highly antioxidative [183]. Moreover, the contents of dietary flavonoids and the antioxidant activity are concentration-dependent [184,185]. Fresh wine shows a unique nutritive value because it contains a lot of polyphenols and high antioxidant activity [186,187]. Raspberry (*Rubus corchorifolius* L.) seed polyphenols are effective antioxidants [188,189]. In the United States, polyphenols provided by apples account for 33% of the total phenolic content contained in all the fruits consumed [190,191]. They are the main source of dietary polyphenols. In addition, the antioxidant capacity of strawberries, blueberries (*Vaccinium* spp.), cranberries (*V*. *macrocarpon* L.) and other berries have been verified *in vivo*, *in vitro* and in clinical studies [192,193,194,195]. More than that, it has also been found that the antioxidant mechanism can effectively prevent the occurrence of cardiovascular diseases. In recent years, many researchers have found that the polyphenols in apples are also highly antioxidative with the functions of antisepsis, inflammation decrease, prevention of coronary heart disease, anti-tumor as well as many other pharmacological functions [196]. Furthermore, Some literatrues reported that cocoa polyphenols could inhibit cytokinin-induced T cell proliferation and prevent the excessive production of B cells through inhibiting the expression of IL-2mRNA and the generation of IL-2 secretions, indicating that polyphenols have certain immunomodulatory effects [197,198].

## 7. Conclusions

Plant phenolics are secondary metabolites that constitute one of the most common and widespread group of substances in plants. To date, plant polyphenols are considered to present a large and diverse array of beneficial effects on both plants and humans. This study presents an overview of oak polyphenols and discusses their basic structures and influencing factors. It also shows the common methods that are widely used for the isolation of phytochemicals, as well as other procedures that enable further progress in the separation and identification of these compounds. Observational studies indicate that oak polyphenols may provide enhancement effects on wine aging systems with a significant improvement in the wine’s color, aroma and taste. Furthermore, oak polyphenols possess various activities, such as anticancer, antioxidant, antidiabetic, antihypertensive, and antimicrobial properties. Although many studies have focused on the oak polyphenols, researchers have failed to provide comprehensive knowledge of their functions during the aging process, such as the mechanism of flavor enhancement and color stability. Moreover, limited information exists on the bioactivity of phytochemicals. For example, what dosage of phenolic compounds can be considered effective for either short-term or long-term effects? Do interactions exist with other nutrients in food? To obtain the answers to the aforementioned questions, further study should be conducted.
